# Characterization of oxytocin and vasopressin receptors in the Southern giant pouched rat and comparison to other rodents

**DOI:** 10.3389/fendo.2024.1390203

**Published:** 2024-05-13

**Authors:** Angela R. Freeman, Samanta Arenas, Danielle N. Lee, Bhupinder Singh, Alexander G. Ophir

**Affiliations:** ^1^ Department of Psychology, Cornell University, Ithaca, NY, United States; ^2^ Department of Biology, Salisbury University, Salisbury, MD, United States; ^3^ Department of Biological Sciences, Southern Illinois University Edwardsville, Edwardsville, IL, United States; ^4^ Comparative Medicine Resources, Rutgers University, New Brunswick, NJ, United States

**Keywords:** autoradiography, social behavior, nonapeptide, social organization, striatum

## Abstract

Vasopressin and oxytocin are well known and evolutionarily ancient modulators of social behavior. The distribution and relative densities of vasopressin and oxytocin receptors are known to modulate the sensitivity to these signaling molecules. Comparative work is needed to determine which neural networks have been conserved and modified over evolutionary time, and which social behaviors are commonly modulated by nonapeptide signaling. To this end, we used receptor autoradiography to determine the distribution of vasopressin 1a and oxytocin receptors in the Southern giant pouched rat (*Cricetomys ansorgei*) brain, and to assess the relative densities of these receptors in specific brain regions. We then compared the relative receptor pattern to 23 other species of rodents using a multivariate ANOVA. Pouched rat receptor patterns were strikingly similar to hamsters and voles overall, despite the variation in social organization among species. Uniquely, the pouched rat had dense vasopressin 1a receptor binding in the caudate-putamen (i.e., striatum), an area that might impact affiliative behavior in this species. In contrast, the pouched rat had relatively little oxytocin receptor binding in much of the anterior forebrain. Notably, however, oxytocin receptor binding demonstrated extremely dense binding in the bed nucleus of the stria terminalis, which is associated with the modulation of several social behaviors and a central hub of the social decision-making network. Examination of the nonapeptide system has the potential to reveal insights into species-specific behaviors and general themes in the modulation of social behavior.

## 1 Introduction

The mammalian neuromodulators oxytocin (OT) and arginine vasopressin (AVP) govern a variety of social behaviors including parental care, affiliation, and aggression, among others (1). These nonapeptides act centrally through their associated receptors, the oxytocin (OTR) and vasopressin 1a and 1b (V1aR and V1bR) receptors. The nonapeptide system is highly conserved, and evolutionary antecedents of OT and AVP are found in birds, amphibians, reptiles, fish, snails, annelid worms and some insects (2–6). Despite the deeply-rooted conservation of the nonapeptide system, differences in the relative density and distribution of OTR and V1aR exist between closely related species (e.g., 7–10). These differences are thought to support species-specific features of behavioral ecology, social organization, and mating tactics (11), and variation within a single species further supports this hypothesis (12–15).

In addition to between- and within-species variation in the distribution and relative density of these receptors, several species exhibit sex differences in receptor density (7, 8, 16–22). These differences between the sexes are region-specific, and are thought to support sex-specific behaviors (16). However, other species lack sex-differences in receptor distribution in OTR or V1aR in the brain (9, 23–25). Not surprisingly, sex differences are only observed if both sexes are studied, and they require measurement of purported sexually dimorphic brain regions. Exploring sex differences in receptor distribution can reveal insight into possible differences in the nonapeptide system sensitivities between the sexes, and provides the essential foundation for functional empirical studies.

To understand the evolutionary trajectory of the nonapeptide system, several research teams have explored how the structure and densities of the nonapeptide receptors differ among species (2, 11, 26, 27). Investigation into the deep homologies of the OT and AVP systems suggest that OTR and V1aR have had different selective pressures leading to variation among species (27), with variation potentially allowing for specialization in behavior within species or genera. As an example, early studies in *Microtus* voles examining the distribution and density of OTR and V1aR receptors suggested monogamy was driven by differences in a few specific brain regions (28). Yet, further work in *Peromyscus* mice showed that variation in receptor density and expression differed between monogamous mice and voles (17, 29). Many taxa will need to be studied to understand how the OT-AVP systems and receptor distribution impacts behavior (30). Despite differences in receptor distribution between genera, such as those found between *Microtus* voles and *Peromyscus* mice, the hypothesis that OT and AVP modulate affiliation and cooperation across rodents and even across mammals has been repeatedly supported (1), suggesting a broad role in the modulation of different ‘flavors’ of social behavior.

The southern giant pouched rat (*Cricetomys ansorgei;* hereafter “pouched rat”) is a large, nocturnal rodent native to sub-Saharan Africa. This species is known for its peculiar reproductive physiology, in which females show profound delays in the development of external genitalia well past ‘adulthood’, and they demonstrate incredible plasticity, such that reproductively active females can revert to a vaginally non-patent (or closed to the outside world) state (31). Furthermore, pouched rats are recognized for their astounding olfactory system and odor discrimination (32–36) and this species has been used as biodetectors for diseases and unexploded ordinances, although it is occasionally mischaracterized as *C. gambianus* (37–39). The pouched rat’s common name is partially based on its convergent rat-like appearance, but they are only distantly related to traditional lab rat species (39, 40). There are very few studies describing the behavior of pouched rats, and fewer describing *C. ansorgei* specifically. However, the aggressive behavior between unfamiliar animals and territorial scent marking behavior (32, 34, 41) suggests that these animals might prefer to live in small family groups. The closely related congener, *C. gambianus*, evidently demonstrates uniparental maternal care, and only one male and female successfully pair and mate when housed in groups of up to six animals (42). Groups of pouched rats have been located together in the wild, but it is unclear how common moderate to large groups are, or if animals found living in groups are related or unrelated (43).

Given the importance of the OT and AVP systems in social behavior, and the need for additional comparative work exploring the evolution of these peptides, we sought to describe the distribution of OTR and V1aR in the brain of the pouched rat (*C. ansorgei*). We hypothesized that differences between the sexes might support sex-specific behaviors in this species like sex differences seen in other rodents. We also hypothesized that the patterning of these receptors’ densities across the social brain might differ from other rodents in ways that mirror the current phylogeny, whereby pouched rats would be most similar to Cricetidae, and potentially also similar to the more distant Muridae.

## 2 Materials and methods

### 2.1 Animals and tissue collection

All work with animals was approved under the U.S. Army Medical Research and Materiel Command (USAMRMC) Animal Care and Use Review Office (ACURO) and the Cornell Institutional Animal Care and Use Committee (IACUC 2014–0043). Tissues were collected from wild-caught animals from Morogoro, Tanzania (6°49’49”S, 37°40’14”E). Prior to collection, animals were housed individually in standard rabbit enclosures and maintained on a 12:12 h light:dark light cycle, at 21°C and 45% humidity. Males and females (assessed by external genitalia) were kept in separate rooms. Animals were fed a standard rodent diet supplemented with dog kibble and fresh fruit and vegetable treats. Chewing bones, a metal ‘stovepipe’ hutch, and dog puzzle toys were given as behavioral enrichment. Newspaper was given for nesting material.

Animals were euthanized via CO_2_ inhalation and brains were swiftly removed and frozen using liquid nitrogen or powdered dry ice and stored at -80°C prior to sectioning. Nine male and eleven female brains were used for this study. Sex was re-assessed and confirmed by gonads at sacrifice. Brains were dissected into blocks coronally by removing the cerebellum, then split sagittally next to the midline into two hemispheres. One hemisphere (preferably the left if unblemished) was coronally sectioned at 20µm thick using a Leica cryostat (CM1950, Leica Biosystems, Nussloch, Germany) set at -20°C. Due to the large size of the pouched rat brains, we mounted every 3rd section and kept six serial sets. Sections were collected from the olfactory bulbs to the start of the cerebellum, and mounted on Superfrost Plus Microscope sides (Fisher Scientific, Pittsburg, PA USA). Microscope slides were stored at -80°C until the autoradiography procedure.

### 2.2 Autoradiography

On two of the sets of slides, we used ^125^I radioligands to label oxytocin receptor (ornithine vasopressin analog, ^125^I-OVTA; NEX 254, PerkinElmer; Waltham, MA) or vasopressin 1a receptor (vasopressin (Linear), V-1A antagonist (Phenylacetyl1, 0-Me-D-Tyr2 [^125^I-Arg6]-); NEX 310, PerkinElmer), as described by Ophir and colleagues (44). Following processing and air-drying, we exposed radiolabeled tissue to film (Kodak Carestream Biomax MR) for 6 days for OTR and 2 days for V1aR to account for differing degrees of decay at the time of use. In each film cassette, we included two ^125^I microscales (American Radiolabeled Chemicals; St Louis, MO), to allow for the conversion of optical density to receptor density. We inferred that receptor density relates to optical density of exposed film, and we therefore used optical measurements as a proxy for receptor density. We digitized films on a Microtek ArtixScan M1 (Microtek, Santa Fe Springs, CA) and measured optical densities using NIH ImageJ Software. We calculated receptor density by first converting optical density to disintegrations per minute (dpm) adjusted for tissue equivalence (TE; for 1 mg in the rat brain), by fitting curves generated by radiographic standards and extrapolating based on these standard curves for each film (see 44).

### 2.3 Quantification and statistical methods

Three sequential sections were measured for density by encircling the regions of interest (ROI) using NIH ImageJ software. The software program calculated mean optical density values and area for ROIs. We measured background labelling by measuring optical density from an area of cortex with no visually-apparent binding in the same section for each ROI. To correctly identify ROIs, we Nissl-stained a third set of tissue to use as a reference, in conjunction with anatomical landmarks identified using a laboratory rat brain atlas. The three measurements for each individual’s ROIs and background were averaged separately, and background was subtracted from the ROI to yield a semi-quantitative measure of receptor density. These final measurements were used for all statistical tests, tables, and figures.

OTR density was measured in the olfactory bulb (OB), anterior olfactory nucleus (AON), prefrontal cortex (PFC), Infralimbic Area (ILA), nucleus accumbens (NAc), caudate-putamen (CP), piriform cortex (Pir), lateral septum (LS), endopiriform cortex (EP), claustrum (CLA), medial bed nucleus of the stria terminalis (BSTm), lateral bed nucleus of the stria terminalis (BSTl), ventral bed nucleus of the stria terminalis (BSTv), ventral pallidum (VPall), medial preoptic area (mPOA), anterior hypothalamus (AH), paraventricular thalamus (PVT), suprachiasmatic nucleus (SCN), paraventricular nucleus of the hypothalamus (PVN), magnocellular hypothalamic nucleus (MCPO), medial habenula (MHb), central amygdala (CeA), medial amygdala (MeA), basolateral amygdala (BLA), ventromedial hypothalamus (VMH), zona incerta (ZIR), lateral hypothalamus (LH), hippocampus (HPC): dorsal CA1, dorsal CA2, dorsal CA3, dentate gyrus (DG), premammilary ventral nucleus (PMV), ventral tegmental area (VTA), periaqueductal gray (PAG), medial geniculate (MG), superior colliculus (SC), and the ventral CA3 of the HPC. V1aR was measured in the same regions except for the MCPO and the MHb. These regions were selected based on work in other species, to allow for comparisons.

To compare receptor densities between sexes, we conducted Welch’s t-tests for each region, to account for unequal variances. We used the highly conservative Bonferroni correction to adjust for multiple comparisons. Test statistics were considered significant when p< 0.05.

We then assessed relative binding using a 4-point scale. Specifically, we used the following definitions: mean OTR< 35 dpm/mg: absent (-), 35 to 490: present (+), 491 to 945: moderate (++), 946 to 1400: dense (+++); mean V1aR< 100 dpm/mg: absent (-), 100–1367: present (+), 1368 to 2634: moderate (++), 2635 to 3800: dense (+++). Values were averaged prior to assessment for regions that had multiple subnuclei (e.g., the BST). These categories are approximated thirds based on overall maximum density, using the methods from Freeman et al. (27). We next used the framework from Freeman et al. (27) to compare overall binding patterns in the pouched rat to those in 23 other species of rodents (27). This framework uses overall OTR and V1aR binding patterns to examine similarities among species, genera, and family groups. Briefly, relative binding patterns within a species were converted to a 4-point scale, using wording and data from previously published studies. These data were then used in a principal components analysis, and species were plotted along the PC1 and PC2 components, with vectors in the plot serving as weights of each variable, and the direction indicating loading on PC1 and PC2. Two plots were created for each receptor type to account for limitations regarding missing data from previous work and for requirements regarding distance matrices when creating these plots. The first plot included as many species as possible and compared among the regions where there were data for all these species. The second plot included as many regions as possible and compared among species that had data for the large number of regions.

We mapped pouched rat relative binding data onto this plot based on this framework, and added data from two recent publications, as available (22, 25; See **Supplementary Data**). In addition to superimposing the pouched rat data onto the PCA biplot, we conducted a comparative permutational MANOVA (‘Adonis2’ function) to examine whether genus or family groups predicted similarities among species’ relative binding patterns using the previously published data from Freeman et al. (27), data from two recent papers (25, 45) and the new data from this paper. All analyses were conducted in R 4.2.1, with the vegan package for the ‘Adonis2’ function, and stats package for t-tests and principal components analysis (46). PCA biplots were made using the ggbiplot function in the ggbiplot package with some aesthetic changes. Data and analysis scripts are available at the Open Science Framework at https://osf.io/6t3cw/.

## 3 Results

### 3.1 Sex differences

Most regions of interest that we investigated showed no significant differences between sexes for OTR or V1aR densities. Although females demonstrated greater OTR density in the superior colliculus compared to males (**Table 1**; t = 2.79, df = 13.58, p = 0.0149), this contrast was no longer significant after corrections for multiple comparisons (adjusted α = 0.001). We detected no significant differences for V1aR density across the brain between sexes (**Table 2**).

**Table 1:** OTR densities by region and sex.

	Females		Males			
Region	Mean Density ± SE (dpm/mg) TE	N	Mean Density ± SE (dpm/mg) TE	N	*t*(df)	*p*
OB	126.3 ± 39.56	9	142.7 ± 39.34	9	-0.30 (16)	0.77
AON	186.4 ± 40.35	9	144.1 ± 30.98	9	0.83 (15)	0.42
mPFC	118.0 ± 36.91	9	94.5 ± 32.08	9	0.48 (16)	0.64
ILA	138.2 ± 43.25	9	72.5 ± 20.32	9	1.37 (11)	0.20
NAc, Core	46.2 ± 14.48	9	37.3 ± 12.45	9	0.46 (16)	0.65
NAc, Shell	81.4 ± 17.96	9	51.8 ± 13.37	9	1.33 (15)	0.20
CP	30.8 ± 13.39	9	23.9 ± 8.42	9	0.43 (13)	0.67
Pir	222.0 ± 39.71	11	192.1 ± 35.8	9	0.56 (16)	0.58
LS	55.2 ± 12.25	11	57.9 ± 13.57	9	-0.15 (17)	0.88
LSd	48.7 ± 7.59	11	56.2 ± 12.33	9	-0.52 (14)	0.61
LSv	71.2 ± 16.63	11	103.5 ± 35.63	9	-0.82 (11)	0.43
EP	147.0 ± 22.53	10	111.8 ± 19.71	9	1.18 (17)	0.26
CLA	192.6 ± 47.69	10	193.2 ± 28.09	9	-0.01 (14)	0.99
BSTm	585.9 ± 117.64	10	532.6 ± 91.23	9	0.36 (16)	0.72
BSTl	687.0 ± 132.48	10	425.7 ± 79.05	9	1.69 (14)	0.11
BSTv	101.6 ± 21.35	10	93.0 ± 28.24	9	0.24 (15)	0.81
VPall	107.3 ± 15.18	10	78.0 ± 23.15	9	1.06 (14)	0.31
mPOA	95.8 ± 19.61	10	88.2 ± 17.61	9	0.29 (17)	0.78
AH	90.3 ± 24.22	10	74.2 ± 17.25	9	0.54 (15)	0.60
PVT	50.9 ± 16.77	10	64.5 ± 27.91	7	-0.42 (10)	0.68
SCN	80.5 ± 23.44	10	125.6 ± 45.86	7	-0.88 (9)	0.40
PVN	14.4 ± 16.18	9	93.9 ± 41.77	7	-1.77 (8)	0.12
MCPO	118.7 ± 37.66	9	122.4 ± 33.6	7	-0.07 (14)	0.94
MHb	545.9 ± 121.71	10	648.4 ± 97.33	9	-0.66 (17)	0.52
CeA	695.2 ± 144.28	9	734.0 ± 92.77	9	-0.23 (14)	0.82
MeA	328.8 ± 51.14	9	381.0 ± 82.29	9	-0.54 (13)	0.60
BLA	627.3 ± 120.17	9	712.9 ± 155.24	9	-0.44 (15)	0.67
VMH	1242.8 ± 245.41	9	1224.4 ± 165.26	9	0.06 (14)	0.95
ZIR	408.5 ± 67.31	10	415.4 ± 58.07	9	-0.08 (17)	0.94
LH	176.0 ± 32.85	10	171.5 ± 41.95	9	0.09 (16)	0.93
dCA1	121.8 ± 25.41	11	107.2 ± 15.49	9	0.49 (16)	0.63
dCA2	106.1 ± 11.57	11	127.5 ± 25.3	9	-0.77 (11)	0.46
dCA3	39.5 ± 10.47	11	45.8 ± 10.29	9	-0.43 (18)	0.67
DG	no binding		no binding			
PMV	536.0 ± 110.84	10	499.3 ± 106.84	9	0.24 (17)	0.81
VTA	524.0 ± 74.13	9	628.2 ± 174.1	9	-0.55 (11)	0.59
PAG	191.2 ± 35.76	10	106.4 ± 28.22	7	1.86 (15)	0.08
MG	no binding		no binding			
SC	199.4 ± 46.21	10	51.5 ± 26.08	7	2.79 (14)	**0.01**
vCA3	51.29 ± 17.71	6	NB	2	2.29 (2)	0.17

Bold value represent a significant difference before adjustment for multiple comparisons. After adjustment, this was no longer statistically significant.

**Table 2:** V1aR densities by region and sex.

	Females		Males			
Region	Mean Density ± SE (dpm/mg) TE	N	Mean Density ± SE (dpm/mg) TE	N	*t*(df)	*p*
OB	1456.5 ± 244.89	9	1841.5 ± 278.59	8	-1.04 (14)	0.32
AON	1287.3 ± 173.38	9	1596.9 ± 235.76	8	-1.06 (13)	0.31
mPFC	588.2 ± 73.53	11	604.4 ± 115.62	9	-0.12 (14)	0.91
ILA	388.2 ± 52.78	11	426.7 ± 111.74	9	-0.31 (12)	0.76
NAc, Core	1319.1 ± 113.88	11	1748.6 ± 266.36	8	-1.48 (10)	0.17
NAc, Shell	1389.3 ± 169.07	11	1687.6 ± 362.54	9	-0.75 (11)	0.47
CP	1725.7 ± 148.69	11	2329.9 ± 322	9	-1.70 (11)	0.12
Pir	604.2 ± 87.76	11	649.1 ± 144.85	9	-0.27 (13)	0.80
LS	3602.8 ± 355.47	11	2885.0 ± 369.04	9	1.40 (18)	0.18
LS, dorsal	3451.2 ± 276.7	11	3613.4 ± 207.77	9	-0.47 (18)	0.65
LS, ventral	2395.9 ± 311.31	11	2351.7 ± 325.22	9	0.10 (18)	0.92
EP	296.6 ± 58.56	11	260.4 ± 57.86	9	0.44 (18)	0.67
CLA	446.1 ± 74.68	11	335.0 ± 58.2	9	1.17 (18)	0.26
BSTm	1552.1 ± 161.04	11	1452.1 ± 229.77	9	0.36 (15)	0.73
BSTl	1297.7 ± 80.59	11	1056.4 ± 188.24	9	1.18 (11)	0.26
BSTv	885.1 ± 108.44	11	835.6 ± 116.42	9	0.31 (17)	0.76
VPall	616.9 ± 104.42	11	470.4 ± 142.24	9	0.83 (15)	0.42
mPOA	626.5 ± 84.5	11	522.3 ± 75.01	9	0.92 (18)	0.37
AH	365.0 ± 97.49	9	601.1 ± 288.18	8	-0.78 (9)	0.46
PVT	63.8 ± 58.22	9	245.1 ± 131.63	8	-1.26 (10)	0.24
SCN	207.9 ± 109.3	9	85.6 ± 71.97	8	0.94 (14)	0.37
PVN	514.6 ± 146.84	8	520.9 ± 161.33	7	-0.03 (13)	0.98
CeA	1710.6 ± 186.92	10	1439.8 ± 188.75	9	1.02 (17)	0.32
MeA	841.82 ± 76.12	10	928.6 ± 160.08	9	-0.49 (12)	0.63
BLA	120.4 ± 66.19	10	139.6 ± 74.36	9	-0.19 (16)	0.85
VMH	390.6 ± 130.5	10	576.4 ± 141.68	9	-0.96 (17)	0.35
ZIR	705.3 ± 78.81	10	738.1 ± 94.39	9	-0.27 (16)	0.79
LH	745.9 ± 91.88	10	915.0 ± 143.4	9	-0.99 (14)	0.34
dCA1	no binding		no binding			
dCA2	119.3 ± 56.35	11	63.4 ± 81.27	9	0.56 (15)	0.58
dCA3	no binding	11	56.0 ± 103.86	9	-1.02 (18)	0.32
DG	281.6 ± 107.99	11	347.8 ± 172.25	9	-0.33 (14)	0.75
PMV	1458.6 ± 333.09	11	2206.8 ± 616.26	9	-1.07 (13)	0.31
VTA	1132.6 ± 186.43	11	1278.4 ± 203.04	9	-0.53 (17)	0.60
PAG	735.0 ± 97.71	11	947.7 ± 293.67	7	-0.69 (7)	0.51
MG	93.9 ± 50.87	11	147.8 ± 117.32	7	-0.42 (8)	0.68
SC	819.1 ± 146.2	11	744.4 ± 229.16	7	0.27 (11)	0.79
vCA3	67.1 ± 78.53	5	119.6 ± 148.52	2	-0.31 (2)	0.79

Bold value represent a significant difference before adjustment for multiple comparisons. After adjustment, this was no longer statistically significant.

### 3.2 Qualitative receptor binding profile

**Figure 1:**
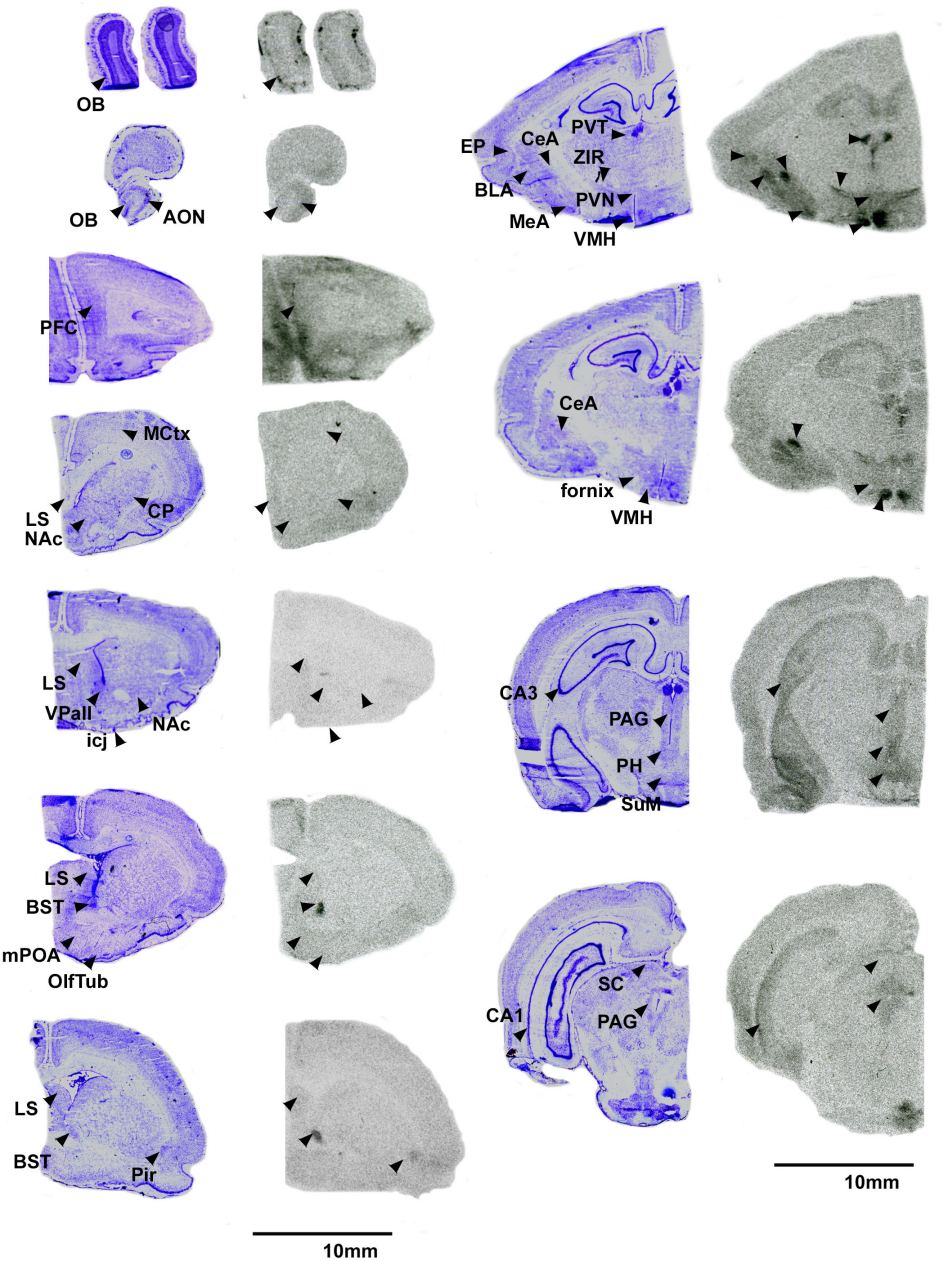
OTR in the pouched rat brain. Images on the left of each pair are Nissl stain, with associated autoradiograms on the right showing OTR density. OB: Olfactory bulb, AON: Accessory olfactory nucleus, PFC: Prefrontal Cortex, MCtx: Motor Cortex, CP: Caudate putamen, LS: Lateral septum, NAc: Nucleus accumbens, VPall: Ventral pallidum, icj: Islands of Calleja, BST: Bed nucleus of the stria terminalis, mPOA: Medial preoptic area, OlfTub: Olfactory tubercle, Pir: Piriform area, PVT: paraventricular thalamus, ZIR: Zona incerta, PVN: Paraventricular nucleus of the hypothalamus, EP: Endopiriform area, BLA: Basolateral amydgala, MeA: Medial amygdala, CeA: Central amygdala, VMH: Ventromedial hypothalamus, CA3: CA3 region of the hippocampus, PAG: Periaqueductal gray, PH: Posterior hypothalamus, SuM: Supramammilary nucleus, CA1: CA1 region of the hippocampus, SC: Superior colliculus. Not shown: VTA: Ventral tegmental area.

Overall, pouched rats had very dense OTR binding in the VMH, with moderate binding in the BST, CeA and VTA (**Table 3**; **Figure 1**). In contrast, we found a low level of binding in the OB and mPFC (**Table 3**, **Figure 1**), and extremely low levels of OTR binding in NAc, LS, PVN, and thalamus (**Figure 1**).

**Table 3:** Relative densities in select regions.

Region	Relative OTR Binding	Relative V1aR Binding
OB	+	++
NAc	+	++
mPFC	+	+
VPall	–	+
LS	+	+++
BST	++	++
CeA	++	++
MeA	+	+
PVN	+	+
HPC	+	–
DG	–	+
PMV	++	++
VMH	+++	+
VTA	++	+

mean OTR < 35 dpm/mg: absent (-), 35 to 490: present (+), 491 to 945: moderate (++), 946 to 1400: dense (+++); mean V1aR < 100 dpm/mg: absent (-), 100-1367: present (+), 1368 to 2634: moderate (++), 2635 to 3800: dense (+++).

**Figure 2:**
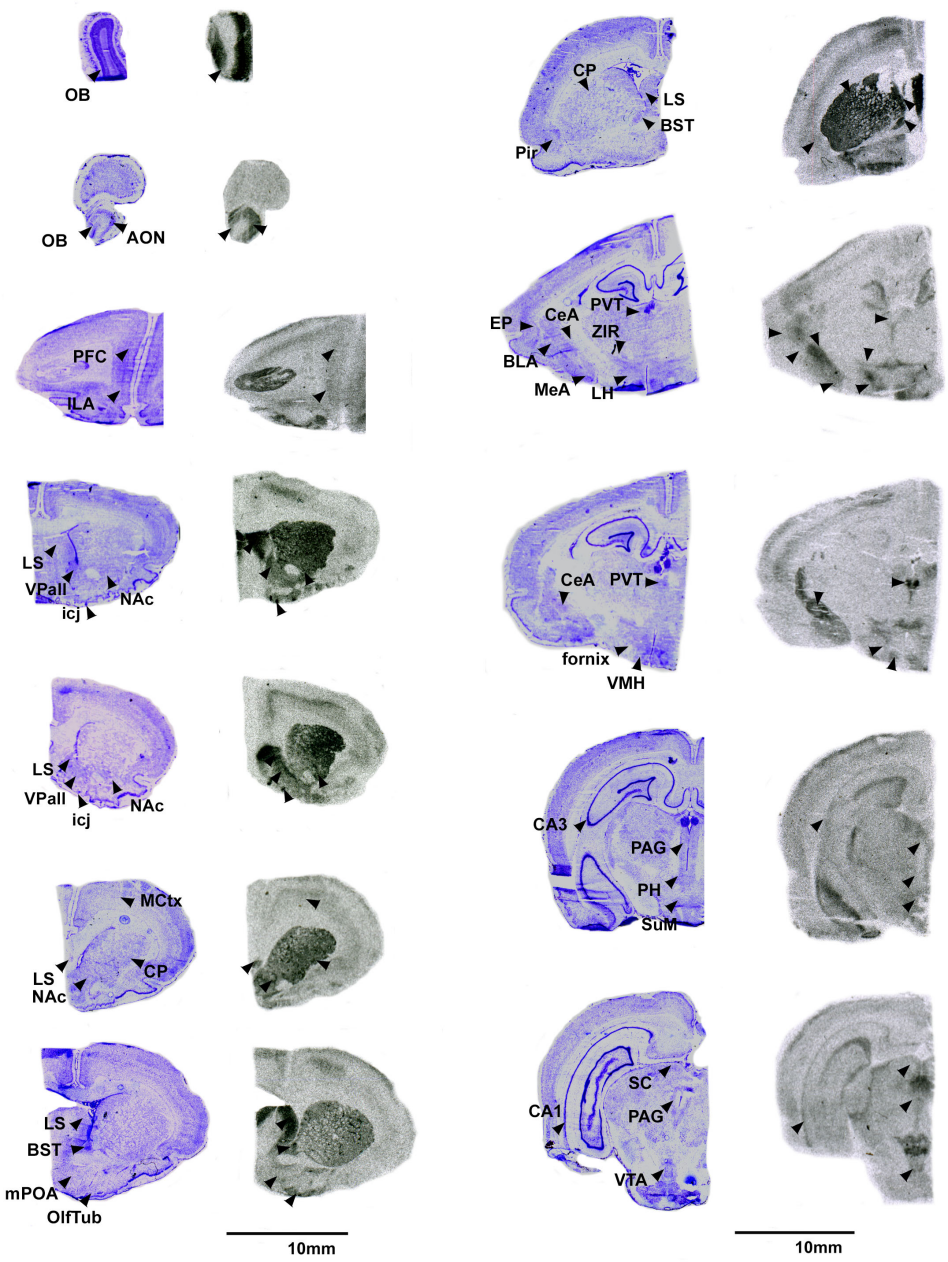
V1aR in the pouched rat brain. Images on the left of each pair are Nissl stain, with associated autoradiograms on the right showing OTR density. OB: Olfactory bulb, AON: Accessory olfactory nucleus, PFC: Prefrontal Cortex, ILA: Infralimbic region of the prefrontal cortex, MCtx: Motor Cortex, CP: Caudate putamen, LS: Lateral septum, NAc: Nucleus accumbens, VPall: Ventral pallidum, icj: Islands of Calleja, BST: Bed nucleus of the stria terminalis, mPOA: Medial preoptic area, OlfTub: Olfactory tubercle, Pir: Piriform area, PVT: paraventricular thalamus, ZIR: Zona incerta, EP: Endopiriform area, BLA: Basolateral amydgala, MeA: Medial amygdala, CeA: Central amygdala, LH: Lateral hypothalamys, VMH: Ventromedial hypothalamus, CA3: CA3 region of the hippocampus, PAG: Periaqueductal gray, PH: Posterior hypothalamus, SuM: Supramammilary nucleus, CA1: CA1 region of the hippocampus, SC: Superior colliculus, VTA: ventral tegmental area.

Pouched rats had relatively very dense V1aR binding in the LS (**Figure 2**), and moderately dense levels of V1aR binding in the olfactory bulbs, BST, NAc, amygdalar nuclei (CeA and MeA), and hypothalamic nuclei (**Figure 2**, **Table 3**). Binding in the HPC was generally absent except for some moderate V1aR binding in the most ventral regions (**Figure 2**, **Table 3**).

### 3.3 Relative receptor binding

We compared pouched rat OTR and V1aR to the overall patterns of binding among 23 (for OTR) and 19 (for V1aR) species of other rodents. To do this, we plotted relative pouched rat OTR and V1aR densities in principal components analysis (PCA)-space with the relative OTR and V1aR densities of the other species for which the same brain regions were available in the published literature. In these PCA biplots, the relative location of a species represents its pattern of binding in the regions identified at the end of the vectors. Therefore, species with similar receptor binding patterns are positioned close together in the plot. The direction of these brain region vectors (i.e., arrows) indicates relative loading on PC1 and PC2, and the length of the vector indicates the weight associated with the two PCs. A species placed near the end of a vector typically indicates relatively dense binding in that region compared to other regions in the plot.

**Figure 3:**
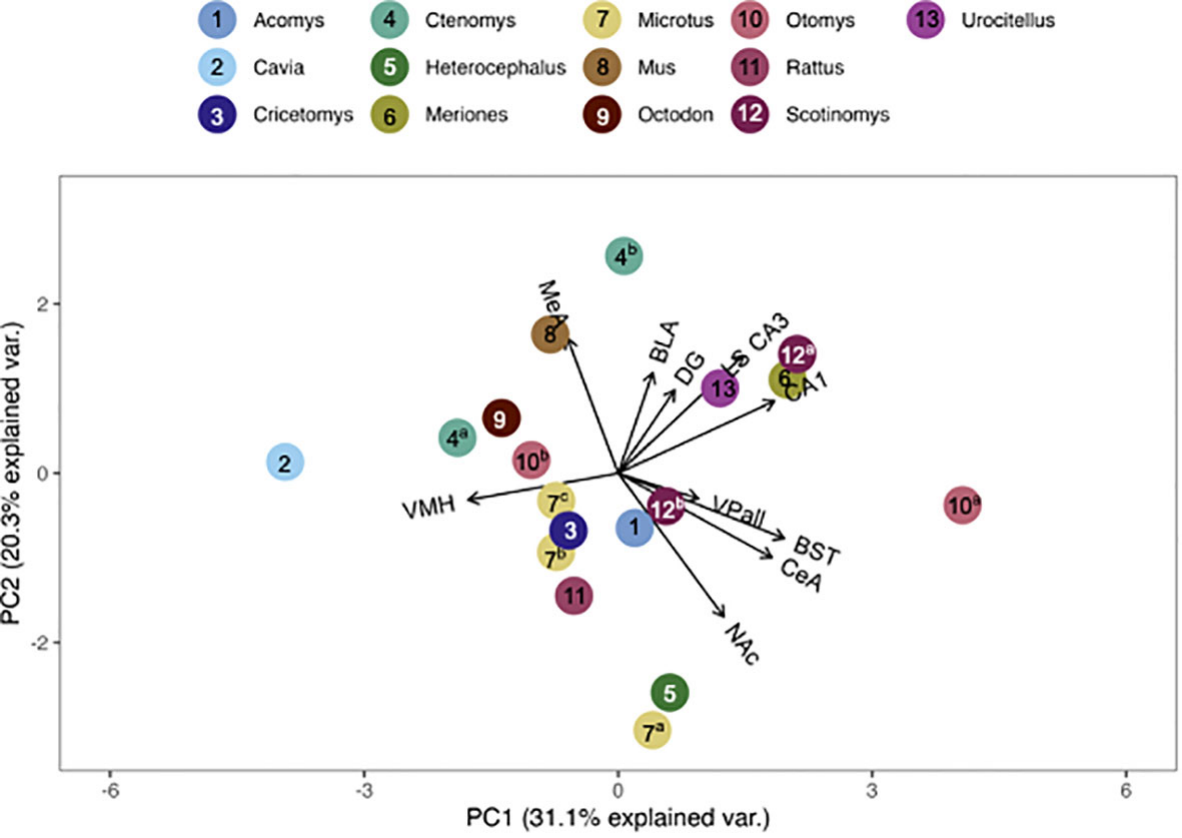
PCA biplot of OTR binding patterns with the number of regions included maximized. Species included in this analysis are 1) *Acomys cahirinus*, 2) *Cavia porcellus*, 3) *Cricetomys ansorgei*, 4a) *Ctenomys sociabilis*, 4b) *Ctenomys haigi*, 5) *Heterocephalus glaber*, 6) *Meriones shawi*, 7a) *Microtus ochrogaster*, 7b) *Microtus kikuchii*, 7c) *Microtus pennsylvanicus*, 8) *Mus musculus*, 9) *Octodon degu*, 10a) *Otomys sloggett*, 10b) *Otomys auratus*, 11) *Rattus norvegicus*, 15a) *Scotinomys xerampelinus*, 12b) *Scotinomys teguina*, 13) *Urocitellus richardsonii*.

**Figure 4:**
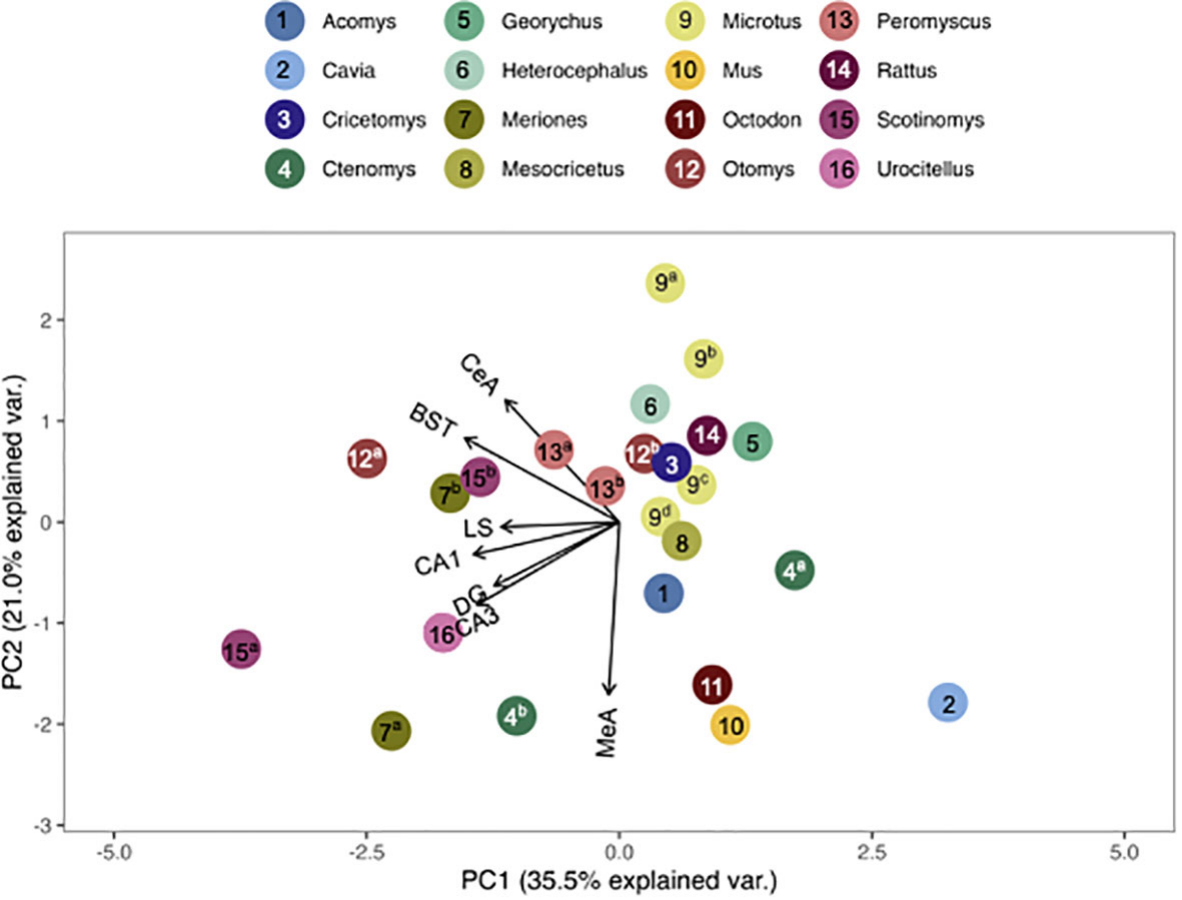
PCA biplot of OTR binding patterns with the number of species included maximized. Species included in this analysis are 1) *Acomys cahirinus*, 2) *Cavia porcellus*, 3) *Cricetomys ansorgei*, 4a) *Ctenomys sociabilis*, 4b) *Ctenomys haigi*, 5) *Georychus capensis*, 6) *Heterocephalus glaber*, 7a) *Meriones unguiculatus*, 7b) *Meriones shawi*, 8) *Mesocricetus auratus*, 9a) *Microtus ochrogaster*, 9b) *Microtus montanus*, 9c) *Microtus kikuchii*, 9d) *Microtus pennsylvanicus*, 10) *Mus musculus*, 11) *Octodon degu*, 12a) *Otomys sloggett*, 12b) *Otomys auratus*, 13a) *Peromyscus maniculatus*, 13b) *Peromyscus californicus*, 14) *Rattus norvegicus*, 15a) *Scotinomys xerampelinus*, 15b) *Scotinomys teguina*, 16) *Urocitellus richardsonii*.

We found that the pouched rat was most similar in OTR binding to *Microtus* voles (**Figures 3**, **4**; figures that include all species names are available in the **Supplementary Data File**). In a comparison that maximized the number of regions included in the analysis, the similarity to *Microtus* was driven by shared low binding in the HPC, including the CA1 and CA3 regions, but relatively high binding in the VMH (**Figure 3**). When the number of species included in the analysis was maximized (**Figure 4**), the pouched rat was placed relatively centrally in the plot, and clustered with several other rodents, suggesting that the overall patterns of OTR binding in the pouched rat brain in the regions that were available for comparison were very similar to most other studied rodents.

**Figure 5:**
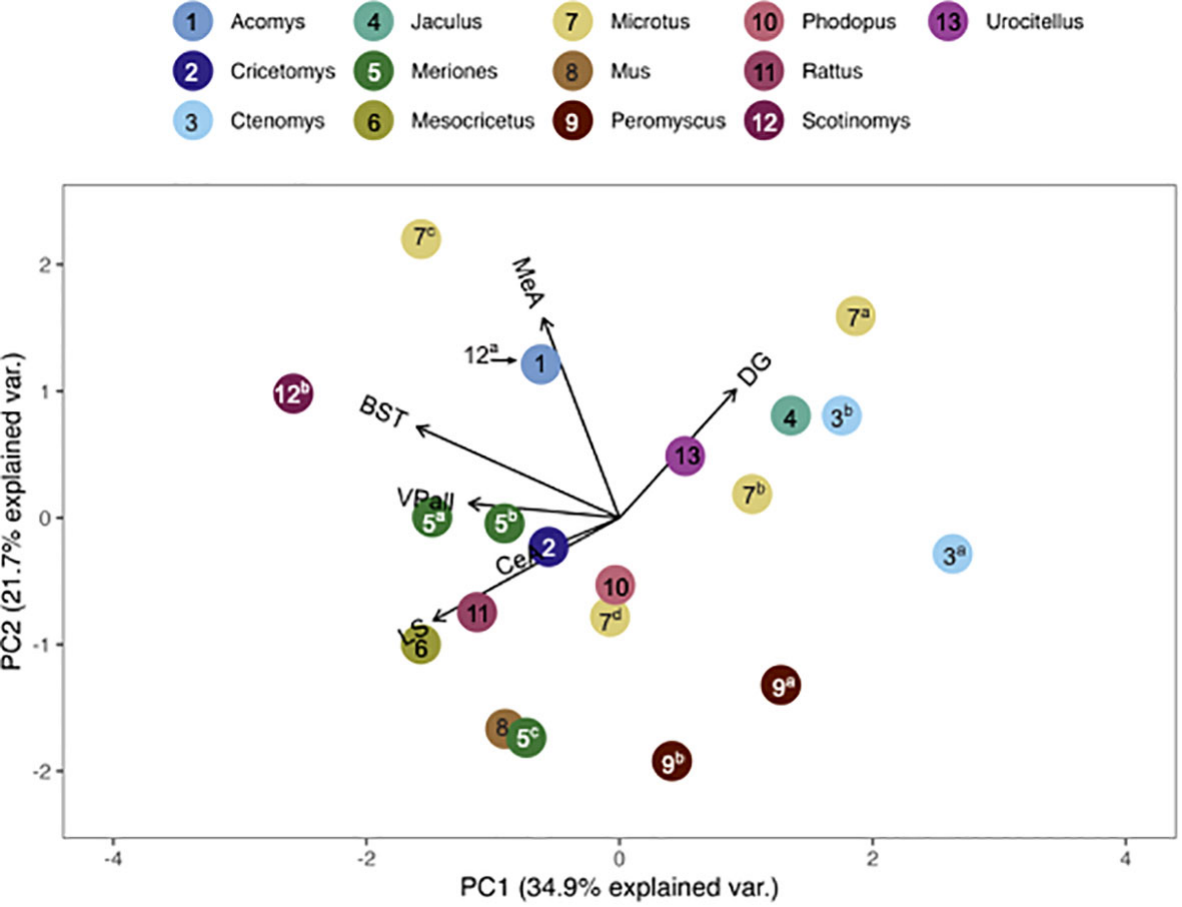
PCA biplot of V1aR binding patterns with the number of species included maximized. Species included in this analysis are 1) *Acomys cahirinus*, 2) *Cricetomys ansorgei*, 3a) *Ctenomys sociabilis*, 3b) *Ctenomys haigi*, 4) *Jaculus orientalis*, 5a) *Meriones shawi*, 5bc) *Meriones unguiculatus*, 6) *Mesocricetus auratus*, 7a) *Microtus ochrogaster*, 7b) *Microtus montanus*, 7c) *Microtus kikuchii*, 7d) *Microtus pennsylvanicus*, 8) *Mus musculus*, 9a) *Peromyscus maniculatus*, 9b) *Peromyscus californicus*, 10) *Phodupus sungorus*, 11) *Rattus norvegicus*, 12a) *Scotinomys xerampelinus* (note, this point fell directly below 1, *A. cahirinus*), 12b) *Scotinomys teguina*, 13) *Urocitellus richardsonii*.

**Figure 6:**
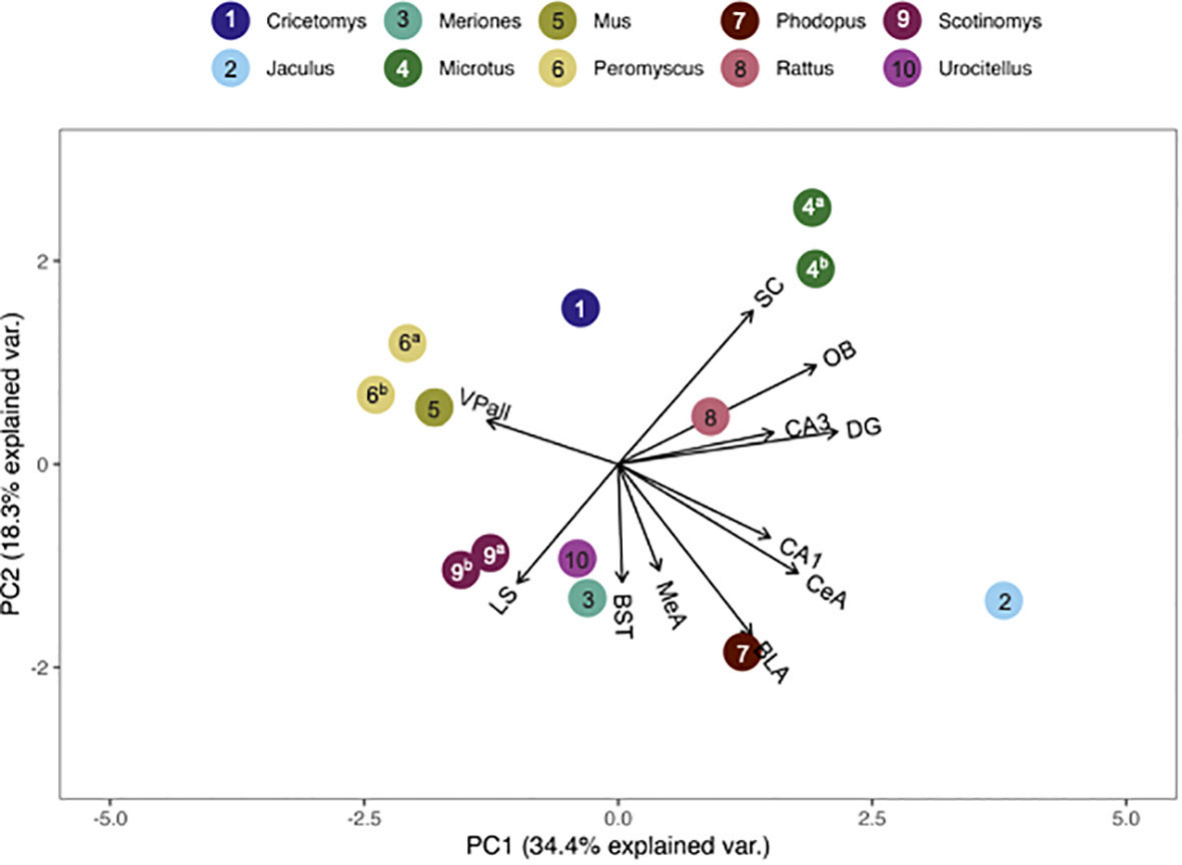
PCA biplot of V1aR binding patterns with the number of regions included maximized. Species included in this analysis are 1) *Cricetomys ansorgei*, 2) *Jaculus orientalis*, 3) *Meriones unguiculatus*, 4a) *Microtus ochrogaster*, 4b) *Microtus montanus*, 5) *Mus musculus*, 6a) *Peromyscus maniculatus*, 6b) *Peromyscus californicus*, 7) *Phodupus sungorus*, 8) *Rattus norvegicus*, 9a) *Scotinomys xerampelinus*, 9b) *Scotinomys teguina*, 10) *Urocitellus richardsonii*.

The pouched rat was placed relatively close to three species of hamsters (*Phodopus sungorus*, *Meriones shawi* and *Meriones unguiculatus*) following the multivariate comparison of V1aR binding, with the number of species included maximized (**Figure 5**). However, a more recent analysis of both male and female *Meriones unguiculatus* described more dense binding in the LS, which differentiated this set of data from the pouched rat (25). This difference may be due to the inclusion of both sexes in the recent work, instead of only males in the previous study. When the number of regions included was increased, the distance between pouched rats and these hamsters increased (**Figure 6**), indicating that binding patterns in the added regions (i.e., CA1 and CA3 regions of the HPC, and the BLA) differ between hamsters and the pouched rat. In this analysis, which had a maximized number of included brain regions, the pouched rat was positioned closely between *Peromyscus* mice and *Microtus* voles (**Figure 6**).

Genus, but not family, predicted both OTR density patterns (**Table 4**) and V1aR density patterns (**Table 5**) in the permutational MANOVAs following the addition of the pouched rat, Mongolian gerbil, and spiny mouse to the dataset. These results indicated that the between-genus variance was larger than the within-genus variance, which can be visualized in the PCAs. In **Figure 4**, the *Microtus* voles group in PCA space and the *Peromyscus* mice were also tightly grouped. In **Figure 5**, the *Microtus* voles had more spread to their grouping, although the *Peromyscus* mice, *Scotinomys* singing mice, and *Ctenomys* Tuco-tucos were tightly grouped by genus.

**Table 4:** OTR Maximized species Permutational MANOVA.

	F	df	SS	R²	p
Family	2.03	7	1.15	0.52	0.05
Residuals	NA	13	1.05	0.48	NA
Total	NA	20	2.20	1.00	NA
Genus	2.40	13	1.79	0.82	0.01
Residuals	NA	7	0.40	0.18	NA
Total	NA	20	2.20	1.00	NA

**Table 5:** V1aR Maximized species Permutational MANOVA.

	F	df	SS	R²	p
Family	1.68	4	0.38	0.34	0.07
Residuals	NA	13	0.73	0.66	NA
Total	NA	17	1.11	1.00	NA
Genus	2.99	10	0.90	0.81	0.002
Residuals	NA	7	0.21	0.19	NA
Total	NA	17	1.11	1.00	NA

## 4 Discussion

We sought to characterize the distribution of OTR and V1aR in the brain of the pouched rat (*C. ansorgei*). We hypothesized that sex differences might help explain some of the sex-specific behaviors that have been observed in this species, as has been the case for some other rodents. However, we found no evidence for sex differences in either the patterns of OTR or V1aR density. We also hypothesized that the patterning of OTR and V1aR density across the social brain might differ from other rodents in ways that mirror phylogenetic patterns of relatedness. Instead, we found that although the patterns of receptor distribution and relative density were fairly common across rodents, pouched rats appeared to be more similar to *Microtus* voles, than other species of rodents for which nonapeptide receptors have been extensively described. Our study highlights the utility of taking a comparative approach to lay groundwork for understanding brain-behavior relationships.

### 4.1 OTR and V1aR densities do not differ by sex in the pouched rat

We detected no differences by sex in the regions we measured OTR and V1aR, with the exception of a potential difference of OTR density by sex in the SC. However, after a correction for the number of comparisons to reduce the likelihood of a Type I error, this difference was not significant. Sex differences in OTR and V1aR density have been reported in several species of rodents (18, 20–22, 47, 48). Nonetheless, like the pouched rat, some species do not demonstrate sex differences in OTR or V1aR density. In particular, no evidence of a sex difference has been found for V1aR in prairie and montane voles (9), V1aR in the jerboa (*Jaculus orientalis*) (49), OTR and V1aR in Richardson’s ground squirrels (23), or OTR and V1aR in Mongolian gerbils (25). We note that OTR in the dorsal HPC appears to differ between the sexes in prairie voles (50), and V1aR can differ between sexes in prairie voles that experienced different rearing conditions (51). Furthermore, one study of voles reported sex differences (18). Receptor densities are labile and are a potential target for selective pressures, which may explain differences found between studies on the same species (47). Notably, *Microtus* voles and to some extent hamsters show the most similar patterns of nonapeptide receptors with pouched rats, and the apparent lack of sex differences presumably contributed to this similarity. Nevertheless, receptor densities were remarkably variable in some regions we examined within each sex among pouched rats. Considering the reports in other species, it is possible that this variation could be attributable to variation in early life experiences (51). However, we are unable to resolve this hypothesis because the tissue we used was collected from animals originally captured in the wild before being housed in captivity, and therefore we do not know their histories. Despite the intriguing variation in receptor density, the lack of sex differences was somewhat unexpected considering the size dimorphism and other aspects of phenotypic differences between the sexes that are readily observable in this species. Understanding the presence or absence of sex differences in the brain can potentially reveal clues about the source of behavioral variation and the function of neural modulation therein. In this instance, the lack of sex differences suggests that nonapeptides serve a non-reproductive role in the modulation of social behavior and/or brain function.

### 4.2 Comparison to other rodents

#### 4.2.1 Differences in the forebrain: OT or AVP for olfactory discrimination?

We predicted that the pouched rat distributions of OTR and V1aR in the brain would be similar to other rodents. Overall, this prediction was supported. However, we also identified a few regions that demonstrated uncommon binding patterns. Notably, there was relatively little OTR binding in the anterior parts of the forebrain, specifically within the OB and medial parts of the prefrontal cortex (mPFC). These regions typically have dense distributions of OTR (e.g., Guinea pig (52), prairie vole (28), mouse (53), meadow vole (54), rat (52)). The mPFC has been implicated in many things, including social bonding, planning and memory and cognition (55), and receives information from the olfactory bulbs, which are the first pass neural processing areas for olfactory information (56). OT signaling in the olfactory regions is critical for social recognition in rats (57) and mice (58), so a lack of these receptors in these regions in pouched rats was unexpected. Indeed, binding was restricted to only a few locations in the glomerular layer of the main OB (**Figure 1**). In contrast to OTR and OT signaling, we found dense V1aR throughout the OB (**Figure 2**), indicating that AVP likely contributes to pouched rat olfactory information processing in a way that OT may not. The social tuco-tuco (*Ctenomys sociabilis*) also lacks OTR in the OBs (7), which raises interesting questions about the role that OT-OTR binding might play in social recognition. Male tuco-tucos disperse from the natal area, while close female kin share burrows (7), both of which presumably rely on the acute ability to discriminate between individual conspecifics. Whether tuco-tucos rely on AVP signaling in olfactory circuitry instead of OT signaling for social recognition is an open question. Pouched rats are exceptional at olfactory discrimination (32, 35, 37, 38), which also raises questions about what OT-OTR in the olfactory sensory system does and whether (or how) low levels of OTR in the OB and parts of the PFC impact sensory processing. Potentially, AVP-V1aR signaling in the olfactory regions of the forebrain might impact social recognition in the pouched rat (59). Furthermore, these results raise questions about whether these animals (or the sensory processing of olfactory cues in general) employ other signaling mechanisms to modulate olfactory discrimination. Further work regarding nonapeptides and their roles in modulating the olfactory system are merited and could begin to answer questions about how processing sensory information and social behavior interact to shape each other (60).

#### 4.2.2 Differences in the BST-LS

We also found an unusual pattern of relatively light OTR binding in the LS compared to relatively dense binding in the BST. In most rodent species, if OTR density is abundant in the BST, it is also abundant in the LS (e.g., California mouse (17), deer mouse (17), Alston’s singing mouse (8), long-tailed singing mouse (8), Richardson’s ground squirrel (23), colonial ice rat (61), vlei rat (61); but see rat (52)). The pattern of relatively dense binding in the BST but little in the LS has only been reported in two species thus far: the prairie vole (28) and the naked mole-rat (10). Much research has focused on differences of OTR density and different species of voles, and how patterns of nonapeptide receptors are indicative of a specific mating system. Unfortunately, little is known about the mating system of pouched rats, though it seems unlikely that pouched rats demonstrate a socially monogamous mating system such as the one employed by prairie voles (62). On the other hand, coordination between BST-LS neural function might have more to do with how sociable an animal is (e.g., 63–65). Naked-mole rats are highly social (typically characterized as eusocial), and the asynchrony in OTR density in the LS and BST might play an important role in modulating this behavior (10). Again, although the pouched rat social organization is poorly understood, it is note-worthy to highlight that pouched rats (*Cricetomys*) and mole rats (*Heterocephalus*) exhibit similar reproductive flexibility based on the social environment (31, 66).

#### 4.2.3 Patterns of binding in the VMH and amygdala

Other regions that showed interesting OTR density patterns included the VMH and the amygdalar nuclei, which had relatively dense binding. The VMH had the densest OTR binding compared to all other regions in the pouched rat. We also detected binding in all of the amygdala subnuclei. OTR is commonly found in these regions in other rodents (27), although binding in the BLA is less common than in central or medial nuclei. OTR binding in the VMH is highly-variable among rodents and explains a large amount of brain-wide pattern variance of OTR (27; **Figure 3**). The VMH is well-known for its regulation of female sex behavior (67), and OTR in this region is modulated by gonadal steroids (68, 69). Males typically have denser binding than females when sex differences of OTR binding have been observed in the VMH (7, 20, 21, 69), although exceptions exist (70) and may be related to steroids during development and adulthood (20, 69). Unexpectedly, both male and female pouched rats had similarly dense OTR binding in the VMH and throughout the brain. It is unknown whether developmental effects or hormonal cycling in the pouched rat might have impacted this OTR phenotype. Given the unique reproductive biology where some females can remain reproductively quiescent for years (31, 33), exploring the interaction between reproductive state and OTR in the VMH may yield insight into the neuroendocrine modulation of this singular phenomenon.

Like most other rodents, we found that pouched rats had V1aR binding in the central and medial amygdala, but little to no binding in the BLA. This specific pattern is similar to singing mice (8), and montane, prairie, and meadow voles (9, 71). Notably, most rodents have some binding in at least one amygdalar nucleus (27). V1aR in the MeA in mice is important for processing social stimuli that enables social recognition (72), largely due to the input from the olfactory system (59). AVP signaling in the CeA, however, is important in maternal aggression (73). Amygdalar AVP in the pouched rat is likely to play a role in processing salience of affect in relation to social stimuli (74), however its role in specific behaviors in the pouched rat requires empirical study. Understanding the role of V1aR in the amygdala and how it interacts with other neural systems has great value because of the multifunctional roles this heterogenous neural structure has and the commonality of V1aR within it.

#### 4.2.4 Comparisons between V1aR and OTR

It is common to find more areas of the brain expressing V1aR than OTR, and pouched rats showed this pattern as well. Like many other rodents, pouched rats had V1aR binding in the olfactory and accessory olfactory systems, LS, BST, NAc, amygdala, and VPall. Perhaps most striking was the dense V1aR binding throughout the CP (caudate putamen, **Figure 2**). No other rodent to date has shown uniform V1aR binding in the striatum (i.e., CP). Indeed, the only other known rodent with V1aR in the CP is *Meriones shawi*, for which binding is restricted to the ventromedial portion of the striatum (75). In *M. shawi*, V1aR is localized to a number of other striatopallidal regions including the VPall, NAc, fundus striati (fStr), and amydalostriatal transition area (aStr) (75). Although we did not quantify V1aR binding in the fStr and aStr separately, we did observe V1aR binding in these regions of the pouched rat striatum. Notably, patterns of V1aR binding in the pouched rat and *M. shawi* are also similar in the EP, the lateral hypothalamus, the PVT, and the CeA. The main difference between these two species seems to be that pouched rats have relatively more V1aR binding in the VMH, whereas *M. shawi* has relatively more binding in the HPC compared to other regions in the *M. shawi* brain.

#### 4.2.5 Extensive V1aR binding across the striatopallidum

Unlike most rodent species but like the pouched rat, the coppery titi monkey (*Plecturocebus cupreus*) also exhibits receptors throughout the striatum (76). Although the function of V1aR in the striatum has not been directly or extensively studied, OTR in the striatopallidum (i.e., NAc and VPall) in prairie voles is important for the modulation of pairbonding behaviors (77). Freeman et al. (76) suggest that V1aR might have a similar function in the pair bonding of titi monkeys, though additional empirical work is needed to know if striatal V1aR contributes to pair bonding. Some work with the Gambian pouched rat (*C. gambianus*) suggests that only one pair of animals within small mixed-sex groups will breed, forming what appears to be a socially-monogamous bond (42). If true and generalizable to *C. ansorgei*, for which the social organization and mating system are unknown, the abundance of V1aR throughout the striatopallidum could potentially contribute to selective affiliative behaviors, and would be consistent with the aforementioned work in new world monkeys. Obviously, much more needs to be done to explore this speculative hypothesis.

The role of V1aR in the VTA is largely understudied, despite the presence of vasopressin or vasotocin immunoreactivity in nearly all vertebrates (47, 78). However, the VTA projects to the NAc, and this circuit is well-recognized for its role in modulating reward, particularly due to the dopaminergic neuronal projections from VTA to the NAc (79). Indeed, the presence of V1aR in the VTA has been previously identified in Syrian hamsters (80) and in low levels in both prairie and montane voles (9). Furthermore, AVP’s action in the VTA can impact self-grooming behavior among Syrian hamsters, although OT appears to be the main modulator of social reward in the VTA of the hamster (80). Whether AVP interacts with the mesolimbic reward system in pouched rats is an open question, yet the presence of receptors in the VTA, CP, NAc and VPall would certainly allow for interactions to occur.

#### 4.2.6 The periaqueductal gray

Finally, we detected V1aR binding in the PAG, which is similar to other rodents, including voles, rats, hamsters, singing mice, and gerbils (8, 9, 20, 25, 71, 75, 81, 82). The PAG is part of, and reciprocally connected to other regions of, the social behavior neural network, which has been touted as modulating social behavior (83, 84). The specific role of AVP-V1aR signaling within the PAG is largely unknown, however injections of AVP in the PAG are known to increase flank marking in hamsters (85, 86). Further work elucidating the role of AVP in the PAG is needed in rodents.

Overall, the pouched rat receptor binding patterns were most like hamsters and voles, although dense V1aR binding in the striatum and the absence of OTR binding in the olfactory nuclei are somewhat unique features among previously published work in rodents.

### 4.3 Limitations

Using wild-caught animals has several advantages for studies such as this one, including providing a description of the natural variation that exists in native outbred populations. Unfortunately, there are also inherent limitations. For example, we do not know the precise age of these animals, their previous reproductive status, or the experiences they had while living in the wild. Therefore, we cannot know whether variables such as these, which have been demonstrated to impact receptor density in other species (20, 87, 88), could have impacted relative densities of receptors in our sample. The data used in the cross-species comparison was also collapsed over sex, which could conceal potential influences of sex on overall OTR and V1aR patterns. Furthermore, receptor distribution and density is an important part of the nonapeptide system, but other differences in this system can explain how behavior is modulated, or what the function of such brain-behavior relationships might be (89–91). It is sometimes assumed that differences in density are biologically meaningful or support functional differences; however, these assumptions are rarely tested directly. Indeed, receptor distribution and density are thought to underlie sensitivity to nonapeptides, peptide release, and neuronal innervation (27, 84, 92). Furthermore, connectivity can explain differences in the function of the OT-AVP system (92). However, site-specific manipulations cannot be conducted without a description of the distribution of these receptors. Therefore, despite the potential limitations of the current report, the extensive description of the distribution of V1aR and OTR in the Southern giant pouched rat provides a foundation upon which important inferences about neuronal modulation and behavioral function can be made. This is strengthened by the growing account of nonapeptide receptor distribution and function across a range of species, which provides a growing comparative perspective of nonapeptide distribution among rodents (30). Thus, this study contributes to insights into the conservation and plasticity of these neuropeptide systems.

## 5 Conclusion

In summary, the Southern giant pouched rat has V1aR and OTR binding patterns that appears to share many common features as those found in hamsters and voles. Uniquely, the pouched rat has strikingly extensive V1aR binding throughout the striatum (i.e., the caudate-putamen), which might function to modulate affiliative behaviors, movement, or other aspects of function for which the striatum is known. Although the biological function for the distribution of these receptors in this species is presently based on what is known from other rodent species, this characterization of the presence and density of V1aR and OTR is essential for providing a more comparative and comprehensive understanding of the evolution of the nonapeptide system and how it supports social behavior.

## Data availability statement

The datasets presented in this study can be found in online repositories. The names of the repository/repositories and accession number(s) can be found below: https://osf.io/6t3cw/.

## Ethics statement

The animal study was approved by Cornell Institutional Animal Care and Use Committee U.S. Army Medical Research and Materiel Command Animal Care and Use Review Office. The study was conducted in accordance with the local legislation and institutional requirements.

## Author contributions

AF: Conceptualization, Data curation, Formal analysis, Investigation, Methodology, Visualization, Writing – original draft, Writing – review & editing. SA: Data curation, Investigation, Methodology, Writing – original draft, Writing – review & editing. DL: Conceptualization, Investigation, Resources, Writing – review & editing. BS: Investigation, Resources, Supervision, Writing – review & editing. AO: Conceptualization, Funding acquisition, Methodology, Project administration, Supervision, Visualization, Writing – original draft, Writing – review & editing.
