# Supplementary material for: Characterization of oxytocin and vasopressin receptors in the Southern giant pouched rat and comparison to other rodents
Source: Front Endocrinol (Lausanne). 2024 May 13;15:1390203. doi: 10.3389/fendo.2024.1390203 (PMC11128605; doi:10.3389/fendo.2024.1390203)

Supplemental data used in Comparative Work

|  | *Meriones unguiculatus* | *Acomys cahirinus* |
| --- | --- | --- |
|  | Mongolian Gerbil | Spiny Mouse |
| Age and Sex | Adult ♀♂ | Adult ♀♂ |
| OB | ND | ND |
| mPFC | + (Figure 3) | ND |
| NAcc | + “light” ~22 | +++ ~112 |
| LS | + “relatively light” ~64 | + ~38 |
| VPall | ND | ++ ~58 |
| BST | ++ ~247 | ++ to +++ 70-112 |
| VMH | + (Figure 3) | ++ ~55 |
| mPOA | + “light” ~39 | + ~46 |
| PVN | + ~43 | ND |
| CeA | ++ ~376 | + (Fig 2) |
| MeA | +++ (Figure 3) | ++ ~82 |
| BLA | +++ “dense” ~530 | ++ ~83 |
| CA1 | +++ (Figure 3) | + ~40 |
| CA3 | +++ (Figure 3) | + ~40 |
| DG | +++ (Figure 3) | + ~40 |
| AH | ++ “moderate ~91 | ++ ~89 |
| PVT | ND | ++ (Fig 2) |
| VTA | + “light” ~13 | ND |
| PAG | ND | - “not detected” |
| MG | ND | ND |
| SC | ND | ND |
| DR | ND | ND |
| Reference | Taylor et al. 2023  Numbered categories: Light = 0 - 91, Moderate = 91* - 470, Dense = 470 - 700  Number values obtained using automeris.io  [automeris.io/WebPlotDigitizer](https://automeris.io/WebPlotDigitizer)  *”Moderate” in text describes values of 91 grayscale. | Powell et al. 2022  Numbered categories: Light = 0 - 47, Moderate = 48 - 92, Dense = 93 - 140  Number values obtained using automeris.io  [automeris.io/WebPlotDigitizer](https://automeris.io/WebPlotDigitizer) |

**OTR**

**V1aR**

|  | *Meriones unguiculatus* | *Acomys cahirinus* |
| --- | --- | --- |
|  | Mongolian Gerbil | Spiny mouse |
| Age and Sex | Adult ♀♂ | Adult ♀♂ |
| OB | ND | ND |
| mPFC | ND | ND |
| NAcc | + “light” ~28 | - “not detected” |
| LS | +++ ~400 | ++ ~57 |
| VPall | + (Figure 3) | ++ ~97 |
| BST | ++ ~144 | ++ ~82 |
| VMH | ND | +++ ~134 |
| mPOA | ++ “moderate ~272 | ++ ~57 |
| CeA | +++ ~388 | + (Figure 3) |
| MeA | - (Figure 3) | ++ ~101 |
| BLA | +++ “dense” ~561 | - “not detected” |
| PVN | ~172 | ND |
| CA1 | - (Figure 3) | - “not detected” |
| CA3 | - (Figure 3) | - “not detected” |
| DG | - (Figure 3) | - “not detected” |
| AH | ++ “moderate” ~161 | ++ ~86 |
| PVT | ND | ++ (Figure 3) |
| VTA | +++ “dense” ~288 | + (Figure 3) |
| PAG | ++ (Figure 3) | ++ ~86 |
| MG | - (Figure 3) | ND |
| SC | - (Figure 3) | +++ (Figure 3) |
| DR | ND | ND |
| Reference | Taylor et al. 2023  Numbered categories: Light = 0 - 100, Moderate = 100-287, Dense = 288* - 900  Number values obtained using automeris.io  [automeris.io/WebPlotDigitizer](https://automeris.io/WebPlotDigitizer)  *”Dense” in text describes values of 288 grayscale. | Powell et al. 2022  Numbered categories: Light = 0 - 55, Moderate = 56-110, Dense = 111-160  Number values obtained using automeris.io  [automeris.io/WebPlotDigitizer](https://automeris.io/WebPlotDigitizer) |

**Supplementary Figures**

**Alternate Figure 3:** PCA biplot of OTR binding patterns with the number of regions included maximized.


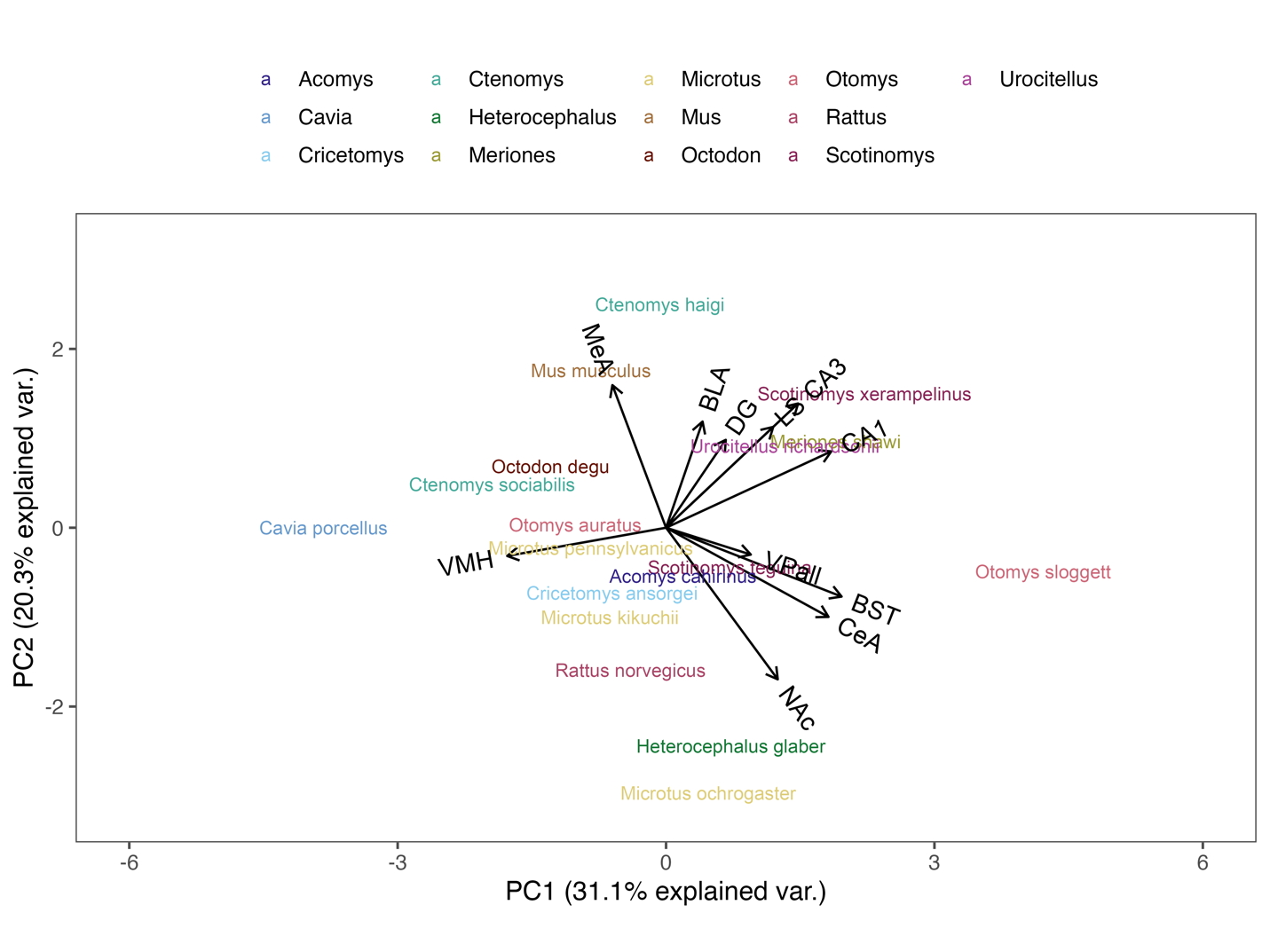


**Alternate Figure 4:** PCA biplot of OTR binding patterns with the number of species included maximized.


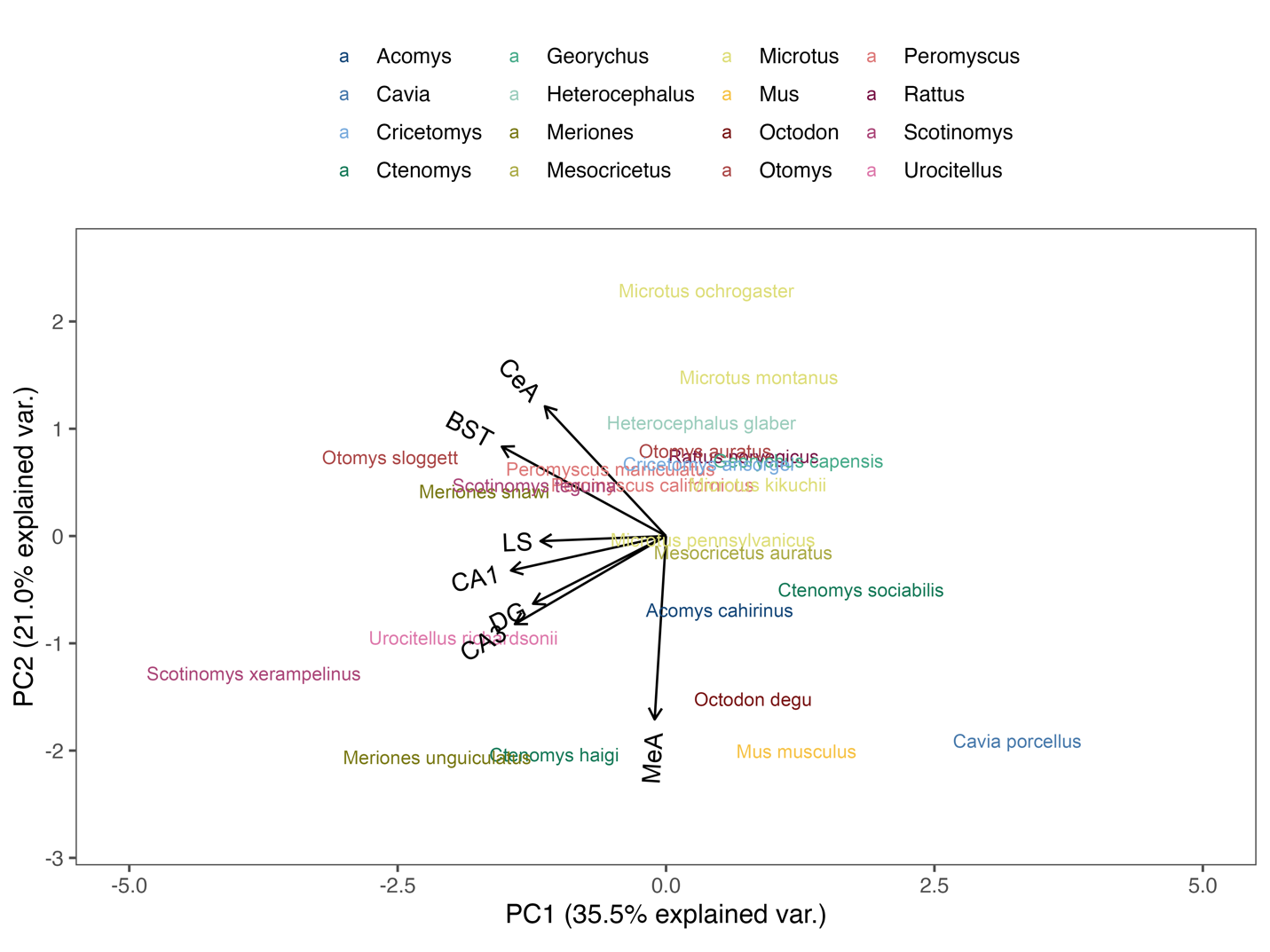


**Alternate Figure 5:** PCA biplot of V1aR binding patterns with the number of species included maximized.


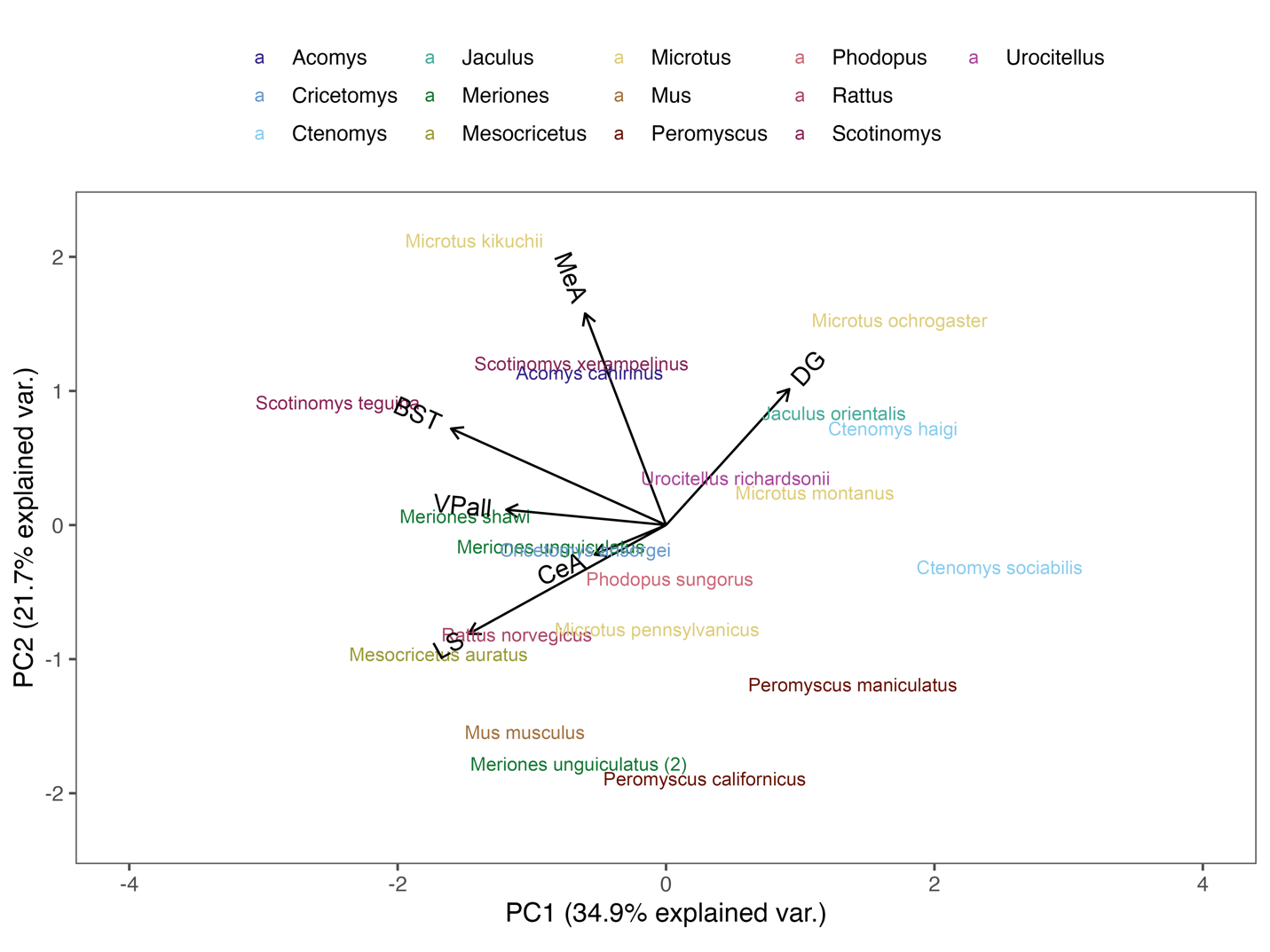


**Alternate Figure 6:** PCA biplot of V1aR binding patterns with the number of regions included maximized.


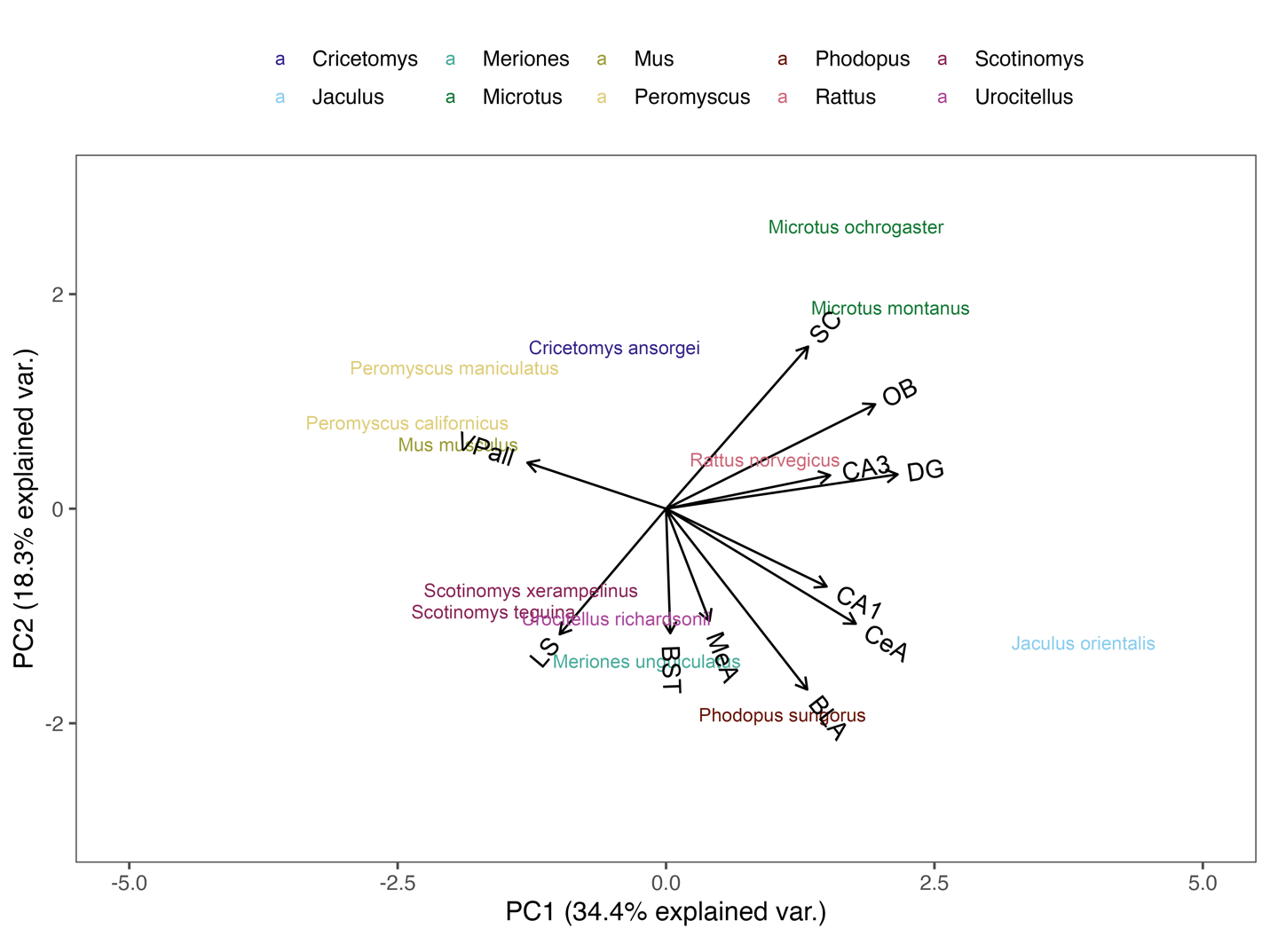

Supplement: Supplementary file 1 [file DataSheet_1.docx]
